# The relationship between atherosclerotic disease and relapse during ATD treatment

**DOI:** 10.3389/fcvm.2022.1039829

**Published:** 2022-10-28

**Authors:** Xinxin Zhu, Yaguang Zhang, Xiaoyu Zhao, Xiaona Zhang, Zixuan Ru, Yanmeizhi Wu, Xu Yang, Boyu Hou, Hong Qiao

**Affiliations:** Department of Endocrinology and Metabolism, The Second Affiliated Hospital of Harbin Medical University, Harbin, China

**Keywords:** atherosclerotic disease, Graves' disease, antithyroid drug, risk factors, drug reduction

## Abstract

**Background:**

Clinical relapse is a potential risk for traditional antithyroid drug (ATD) treatment in hyperthyroid patients. Evidence suggests that atherosclerotic disease is closely associated with hyperthyroidism, while the relationship between atherosclerosis and relapse remains unclear.

**Methods:**

Two hundred and twenty-five patients with GD who underwent ATD as their first treatment were studied; 88 and 137 patients were categorized as drug reduction relapse and drug reduction remission, respectively. Logistic regression was used to analyze risk factors of drug reduction relapse in patients with GD.

**Results:**

During a median of 48 months followed up 88 patients who relapsed. According to multivariate analyses, atherosclerosis related diseases, FT4, goiter, and anxiety rating scores are independent risk factors for drug reduction. According to K-M survival analysis, patients with atherosclerosis related diseases, FT4 > 18.82 pmol/L, anxiety rating scores > 23, and gradation of goiter ≥ Grade II had a higher risk of relapse than those with lower levels. ROC analysis shown atherosclerosis related diseases significantly improved the predictive accuracy of relapse.

**Conclusions:**

Atherosclerotic disease is closely related to the relapse of hyperthyroidism, ATD treatment in hyperthyroid patients with atherosclerosis should be given more attention.

## Introduction

As one of the most common endocrine diseases, hyperthyroidism is characterized by increased synthesis and secretion of thyroid hormone. Clinically, Graves' disease (GD) represents the most common form of hyperthyroidism, accounting for 50–80% of all cases (1, 2), and the incidence of GD has been increasing over the past few years (1, 3). Over 30% of patients with GD suffered from Graves' orbitopathy (GO) (4–6), which caused vision loss (7). Additionally, it has been reported that GD was associated with the incidence of major adverse cardiac events (MACE) (8, 9). Atherosclerosis is a chronic inflammatory disease. In addition, there is also evidence that chronic inflammation contributes to hyperthyroidism, which suggests a potential relationship between atherosclerosis and hyperthyroidism. Patients with hyperthyroidism have more high-grade coronary stenosis, plaque burden and high-risk plaque characteristics, which suggested that elevated thyroid hormone can lead to coronary artery vascularization and plaque instability (10). However, the impact of atherosclerotic diseases on the treatment and prognosis of hyperthyroidism remains unclear.

Antithyroid drugs (ATDs) are the first line treatment for patients with GD and contribute to the remission of clinical symptoms, supported by substantial clinical trials with a positive outcome (11–14). According to the current guidelines (3, 15, 16), it is recommended to decrease the dose to 2.5–10 mg/d or 100–150 mg/d after starting with a higher dose of Methimazole (MMI) (10–20 mg/d) or Propylthiouracil (PTU) (150–300 mg/d) after thyroid function returns to normal. However, some studies have shown that certain patients with GD relapsed during drug reduction (17–20). Hence, in order to achieve individualized treatment for patients with GD, it is necessary to study the independent risk factors of relapse in those with drug reduction.

Herein, we conducted this study to explore the effect of atherosclerosis related diseases on drug reduction and recurrence in patients with hyperthyroidism, which will facilitate our understanding of risk stratification and management in patients with GD.

## Method

### Study population and design

This study is a retrospective cohort study abiding by the Declaration of Helsinki. The whole research process was authorized and supervised by the Second Affiliated Hospital of Harbin Medical University ethics committee. All experimental subjects completely understood the research process and signed informed consent forms, which encompassed data use and follow-up.

**Figure 1:**
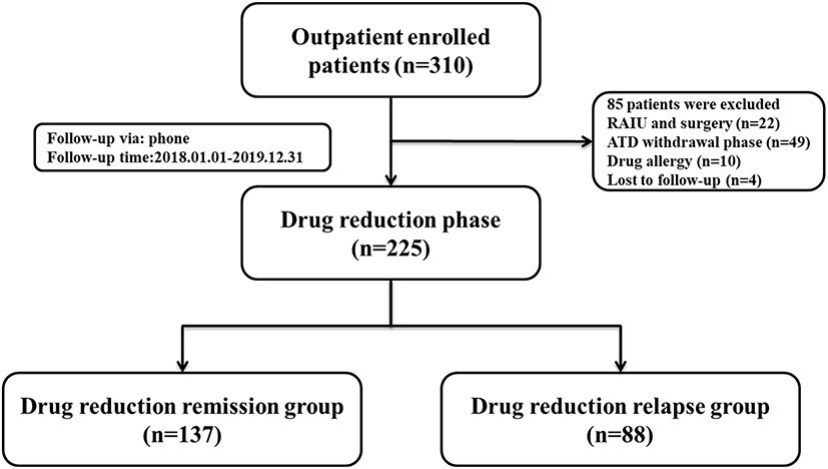
Flowchart of the study population enrollment. ATD, antithyroid drug; RAIU, radioactive iodine ablation.

In accordance with the current guidelines (3, 16), 310 patients with GD from the Second Affiliated Hospital of Harbin Medical University were enrolled between January 1, 2018 and December 31, 2019. Patients who met the following criteria were included: (1) Patients identified with GD. (2) Patients in the process of standardized ATD treatment. (3) Patients without radioactive iodine ablation (RAIU) and surgery. (4) Patients who signed informed consent and were informed about the study. Meanwhile, patients who met any of the following criteria were excluded from this study: (1) Patients with severe hyperthyroidism complications. (2) Patients with drug and food allergy. (3) Patients who were still participating in other clinical trials. (4) Patients who could not take medicine as prescribed. (5) Patients who were lost to follow-up. (6) Patients who did not return to the prescribed return visit date. (7) Patients with incomplete examination materials. In light of exclusion criteria, 85 patients were excluded. The study flow chart was shown in Figure 1. Finally, data on 225 patients was used to the further analysis with a median follow-up of 48 months (range 16–48 months). Enrolled GD patients were divided into two groups: Drug reduction relapse group (*n* = 88): During ATD treatment, patients with GD were cured or the clinical symptoms were relieved. While after FT3, FT4, and TSH returned to normal levels, the situation of increased FT3 and FT4 appeared again (17–20). Drug reduction remission group (*n* = 137): During ATD treatment, the clinical symptoms were relieved, and FT3, FT4, and TSH returned to normal levels (18–20). Normal ranges of hormones: FT3 (range 2.43–6.01 pmol/L), FT4 (range 9.01–19.50 pmol/L), TSH (range 0.35–4.94 uIU/mL), TRAb (range 0–1.5IU/L).

### Data

Clinical data were collected at the beginning of drug reduction. Patients with GD' gender, age, history of GD, GD genetic history, gradation of goiter, palpation of a hard thyroid, irregular drug use, smoking history, drinking history, anxiety rating scores, complications, Free triiodothyronine (FT3), Free thyroxine (FT4), Thyrotropin (TSH), Thyrotropin receptor antibody (TRAb) level, whether to replace the drugs, and whether to combine with other drugs and other clinical data in every phase were collected. Additionally, hyperthyroidism diagnostic criteria can be listed as follows: (1) Hyper metabolic symptoms and signs. (2) Goiter. (3) Serum TSH level decreased and thyroid hormone level increased. The diagnosis can be made when the above three conditions are achieved. As for GD diagnostic criteria: (1) The diagnosis of hyperthyroidism was established. (2) Diffuse enlargement of the thyroid gland (palpation and ultrasound confirmed). (3) Exophthalmos and other invasive eye signs. (4) Pretibial myxedema. (5) Positive TRAb and Thyroid peroxidase antibody (TPOAb) level. In the above criteria, items (1)-(2) are necessary diagnostic conditions, and items (3)-(5) are auxiliary diagnostic conditions. Definitions are provided in Table 1.

**Table 1:** Definition of clinical baseilne.

**Relapse**	**The clinical features of GD relapse including the presence of conventional symptoms of hyperthyroidism associated with a diffusely enlarged goiter; elevated levels of FT3, FT4 and depressed TSH level (20)**.
**Remission**	**Reliefs of clinical symptoms and maintaining euthyroid state (11)**.
**Treatment and drug reduction**	**Based on the guidelines, MMI was used (30–20–10–5–2.5 mg/d-withdrawal) throughout the entire ATD treatment process (3, 14, 15)**.
**Reduced dosage 10 mg**	**MMI (30–20–10 mg/d)**
**Reduced dosage 5 mg**	**MMI (10–5 mg/d)**
**Reduced dosage 2.5 mg**	**MMI (5–2.5 mg/d-withdrawal)**
**Goiter**	**The abnormal enlargement of the thyroid gland and diagnosed at the time of initial physical examination. It was categorized according to the World Health Organization goiter classification system: Grade 1, thyroid is palpable but not visible when the neck is in the normal position; Grade 2, thyroid is palpable and visible but within the sternocleidomastoid; Grade 3, thyroid is palpable and visible but beyond the sternocleidomastoid (21)**.
**Side effects**	**Common side effects of ATD consists of rash, urticaria, and arthralgia. Meanwhile, rare but major side effects include hepatitis, a lupus-like syndrome, and agranulocytosis (3, 14, 15, 22)**.
**Complications**	**Containing weight loss, osteoporosis, fragility fractures, atrial fibrillation, embolic events, and cardiovascular dysfunction would be more likely to occur (3, 9, 23, 24)**.
**Self-rating Anxiety Scale (SAS)**	**Self-report scale whose 20 items cover a variety of anxiety symptoms, both psychological (e.g., “I feel afraid for no reason at all” and “I feel like I'm falling apart and going to pieces”) and somatic (e.g., “My arms and legs shake and tremble” and “I feel my heart beating fast.”) in nature. Responses are given on a 4-point scale which ranges from 1 (none, or a little of the time) to 4 (most, or all of the time). Participants were instructed to base their answers on their experiences over the last week. Items are concerned with both negative and positive (e.g., “I fall asleep easily and get a good night's sleep.”) experiences, with the latter being reverse scored. Add the rough score of the 20 items and multiply the rough score by 1.25 to get the standard score. The standard score of more than 50 points indicates that anxiety exists, the greater the score value, the more serious anxiety, including 50–59 identified as mild anxiety, 60–69 identified as moderate anxiety, and more than 70 points identified as severe anxiety (25)**.
**Irregular drug use**	**A patient who does not take the medication at a regular time, but takes the daily oral dose prescribed by the doctor**.
**Combined with other drugs**	**Patients who take these drugs to relieve symptoms or complications of GD, such as Propranolol Hydrochloride, Bicyclol Tablets, Leucogen Tablets**.
**Atherosclerotic disease**	**Patients were diagnosed with atherosclerotic diseases such as coronary heart disease, stroke and peripheral vascular disease**.

### Statistical analysis

We tested data normality using the Kolmogorov-Smirnov method. Continuous data are presented as mean ± SD or median (interquartile range). The student test or the Mann-Whitney U test was used for statistical comparisons in two groups. Categorical variables are presented as count (percent), and comparisons between groups were made with the χ2 or Fisher exact test. A logistic regression model was used to evaluate the association between risk factors and drug reduction relapse, which was adjusted for all other baseline characteristics with *P* < 0.10 on univariable analysis. Moreover, Kaplan–Meier (K-M) survival analysis was applied to compare the differences in various baselines between two groups. And receiver-operating characteristic curve (ROC) was performed to evaluate the accuracy that serum FT3, FT4, and TSH level for drug reduction relapse.

## Results

### Baseline data characteristics

Among the 310 patients with GD who initially underwent ATD treatment, 85 patients were excluded because of RAIU or surgery (*n* = 22), after ATD withdrawal (*n* = 49), drug allergy (*n* = 10) and lost to follow up (*n* = 4). The remaining 225 patients were suitable for of drug reduction assessment, according to the current guidelines (15, 16), 137 patients had drug reduction relapse and 88 patients had drug reduction remission. The clinical baseline characteristics for the entire study cohort are displayed in Table 2 based on the clinical data of patients in two groups. The proportion of patients with Grade II and above goiter (94.32 vs. 75.18%, *P* < 0.001), with palpation of a hard thyroid (19.32 vs. 7.30%, *P* = 0.007), with irregular drug use (38.64 vs. 16.06%, *P* < 0.010), smoking patients (32.95 vs. 20.15%, *P* = 0.025), alcohol drinking patients (43.18 vs. 24.82%, *P* = 0.019), incidences of complications (90.91 vs. 75.91%, *P* = 0.001) and side effects (22.73 vs. 9.59%, *P* = 0.006) were significantly higher in relapse group than that in remission group, while the two groups showed no significant differences in gender, age, duration of GD, and GD genetic history. However, it was found that atherosclerotic diseases were significantly higher in the relapsing group than in the remission group.

**Table 2:** Clinical baseline of patients in the drug reduction group.

**Variables**	**Remission group (*n* = 137)**	**Relapse group (*n* = 88)**	***P*** **-value**
Gender (male, %)	28 (20.44%)	23 (26.14%)	0.319
Age (years)	39.31 ± 13.08	39.28 ± 11.80	0.495
Duration of GD (years)	1.88 ± 1.61	2.12 ± 1.72	0.150
GD genetic history (%)	4 (3.01%)	3 (3.41%)	0.837
Gradation of goiter (Degree II and above, %)	103 (75.18%)	83 (94.32%)	<0.001
The palpation of thyroid (hard, %)	10 (7.30%)	17 (19.32%)	0.007
Irregular drug use (%)	22 (16.06%)	34 (38.64%)	<0.001
Smoking history (past or present smokers, %)	27 (20.15%)	29 (32.95%)	0.025
Drinking history (past or present drinker, %)	34 (24.82%)	38 (43.18%)	0.019
Complication (%)	104 (75.91%)	80 (90.91%)	0.001
Atherosclerotic disease	25 (18.2%)	42 (47.7%)	<0.001
Side effect after ATD (%)	13 (9.59%)	20 (22.73%)	0.006

GD, Graves' disease; ATD, antithyroid drug; WHO goiter classification system, Grade I, thyroid palpable but not visible when the neck is in the normal position; Grade II, thyroid palpable and visible but within the sternocleidomastoid; Grade III, thyroid palpable and visible but beyond the sternocleidomastoid.

In addition, the laboratory baselines characteristics of patients were shown in Table 3. Patients in the relapse group had significantly higher anxiety rating scores (30.98 ± 15.97 vs. 23.08 ± 13.15, *P* < 0.001), the serum levels of FT3 (10.07 ± 5.64 pmol/L vs. 6.11 ± 3.90 pmol/L, *P* < 0.001) and FT4 (29.78 ± 15.81 pmol/L vs. 16.64 ± 9.57 pmol/L, *P* < 0.001) than those in the remission group.

**Table 3:** Laboratory baseline of patients in the drug reduction group.

**Variables**	**Remission** **group** **(*n* = 137)**	**Relapse** **group** **(*n* = 88)**	***P*** **-value**
Anxiety rating scores (%)	23.08 ± 13.15	30.98 ± 15.97	<0.001
FT3 (pmol/L)	6.11 ± 3.90	10.07 ± 5.64	<0.001
FT4 (pmol/L)	16.64 ± 9.57	29.78 ± 15.81	<0.001
TSH (uIU/mL)	3.37 ± 6.62	0.60 ± 2.39	<0.001
TRAb (IU/L)	6.16 ± 7.61	7.55 ± 8.59	0.116
MMI (%)	124 (90.51%)	75 (85.23%)	0.226
Combine with other drugs (%)	104 (75.91%)	74 (84.10%)	0.141

FT3, free triiodothyronine; FT4, free thyroxine; TSH, thyrotropin; TRAb, thyrotropin receptor antibody; MMI, Methimazole.

Furthermore, the serum level of TSH (0.60 ± 2.39 uIU/ mL vs. 3.37 ± 6.62 uIU/ml, *P* < 0.001) in the relapse group was lower than that of remission group. However, no differences were observed in the serum level of TRAb, patients with MMI, and whether patients had combined with other drugs between two groups.

### Baselines related to the relapse of Graves' disease

**Figure 2:**
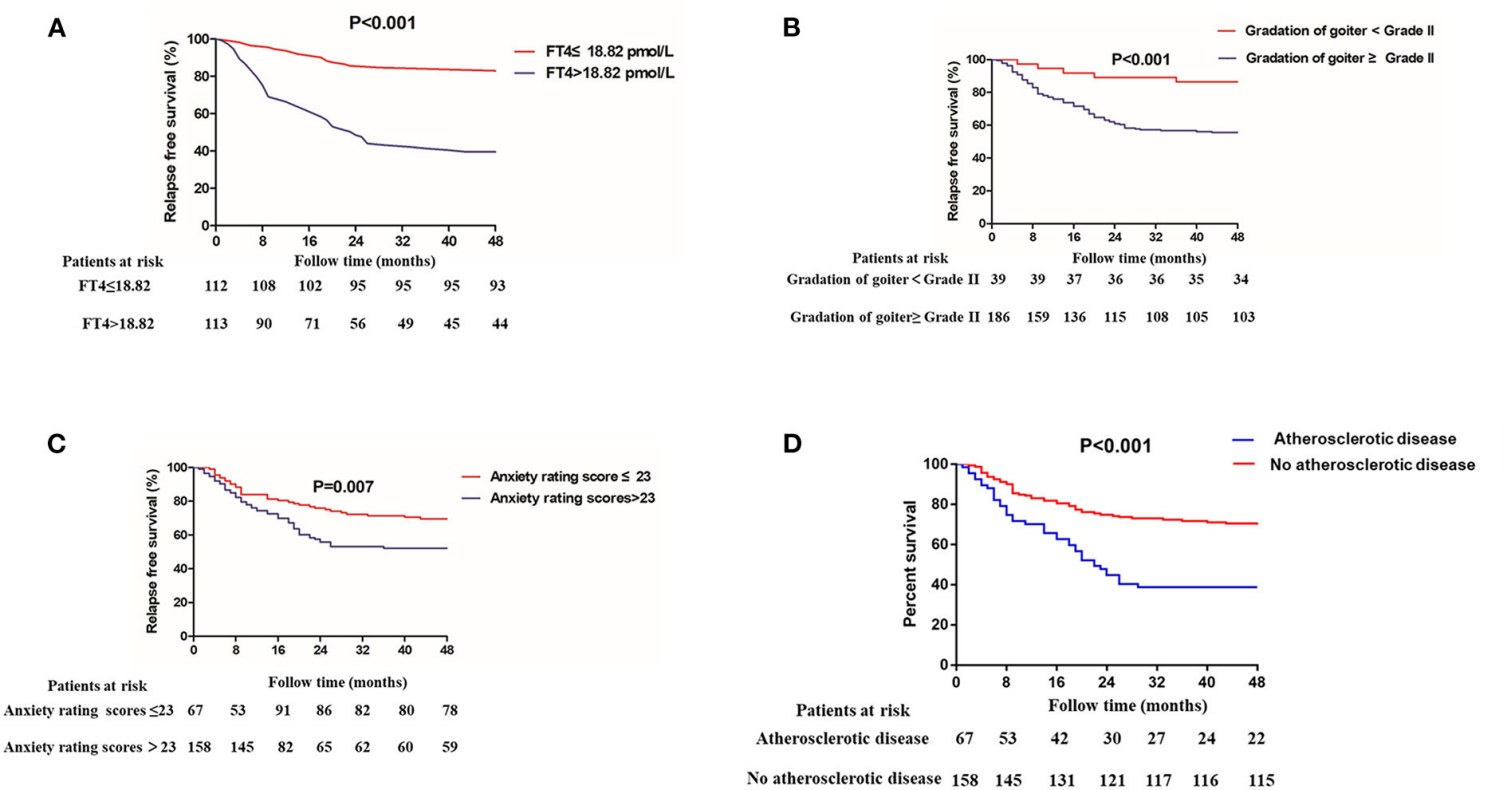
Kaplan–Meier analyses for patients with GD who underwent antithyroid drug (ATD) treatment and relapse during ATD reduction. **(A)** The serum level of free thyroxine (FT4), **(B)** goiter gradation, **(C)** anxiety rating scores, and **(D)** Atherosclerotic disease.

**Figure 3:**
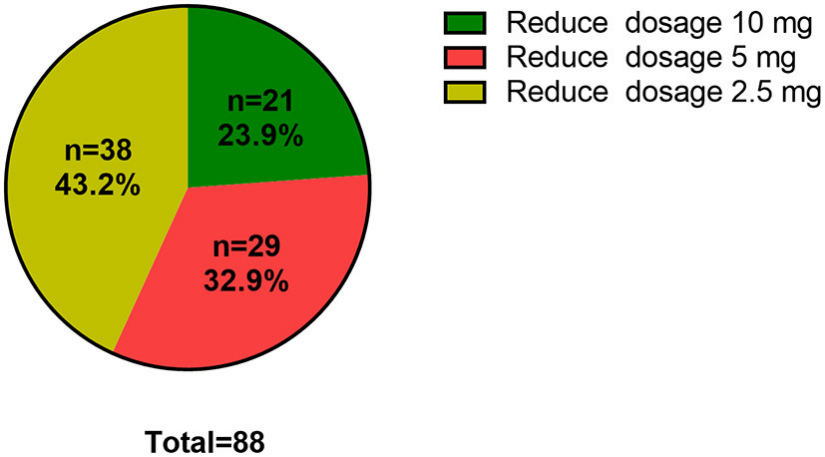
Proportion of relapse in dosage reduction. Reduced dosage 10 mg, MMI (30–20–10 mg/d); Reduced dosage 5 mg, MMI (10–5 mg/d); Reduced dosage 2.5 mg, MMI (5–2.5 mg/d-withdrawal).

During drug reduction, 88 of 225 (39.11%) patients experienced relapse. In order to explore the clinical risk factors of patients who underwent drug reduction relapse, logistic regression was used to analyze the clinical and laboratory baselines of patients between remission group and relapse group. After adjustments for traditional risk factors, the following factors were obviously related with relapse during drug reduction: goiter gradation grade II and above (OR = 4.473, 95% CI = 1.236–16.189, *P* = 0.022), patients with higher anxiety rating scores (OR = 1.044, 95% CI = 1.019–1.069, *P* = 0.001) and higher serum level of FT4 (OR = 1.096, 95% CI = 1.034–1.162, *P* = 0.002) (Table 4). In addition, K-M survival curves were used to further evaluate the association between goiter gradation, anxiety rating scores, atherosclerotic disease, FT4 and drug reduction relapse (Figure 2). Considering the median value of FT4 and anxiety rating scores, the patients were divided into two groups (high FT4 group, FT4 > 18.82 pmol/L, high anxiety rating scores group, anxiety rating scores > 23, low FT4 group, FT4 ≤ 18.82 pmol/L, low anxiety rating scores group, anxiety rating scores ≤ 23). K-M survival curves demonstrated that patients with FT4 > 18.82 pmol/L (*P* < 0.001) and anxiety rating scores > 23 (*P* = 0.007) showed significantly higher RFS than those patients with lower levels. Furthermore, goiter gradation was composed of two groups (high goiter group: Gradation of goiter ≥ Grade II, low goiter group: Gradation of goiter < Grade II). Consequently, patients who had Gradation of goiter ≥ Grade II were associated with higher RFS in patients with GD by K-M survival curves analysis. Patients with atherosclerotic disease showed significantly higher RFS than those patients with lower levels. Furthermore, in order to assess recurrence in different reduced dosage, the reduced dose was divided into three groups in light of the guidelines (Reduce dosage 10 mg, reduce dosage 5 mg, Reduce dosage 2.5 mg). Pie chart showed that Patients with GD who reduce dosage 2.5 mg (43.2%) were more likely to relapse (Figure 3).

**Table 4:** Logistic analysis of risk factors.

**Variables**	**Unadjusted**			**Adjusted**		
	**OR**	**95% Cl**	***P*** **-value**	**OR**	**95% Cl**	***P*** **-value**
Gender	1.377	0.733–2.590	0.320			
Age	1.000	0.979–1.021	0.990			
Duration of GD	1.088	0.927–1.277	0.300			
Gradation of goiter						
Grade I	Ref					
≥Grade II	5.267	1.969–4.088	0.001	4.473	1.236–16.189	0.022
The palpation of thyroid (hard)	3.041	1.322 to 6.997	0.009	0.775	0.278–2.160	0.626
Irregular drug use	3.291	1.760–6.156	<0.001	0.464	0.204–1.055	0.067
Smoking history	2.003	1.086–3.694	0.026	1.094	0.337–3.545	0.881
Drinking history	2.302	1.298–4.083	0.004	0.571	0.186–1.754	0.328
Complications	3.173	1.390–7.245	0.006	0.408	0.136–1.224	0.110
Side effect after ATD	2.805	1.314–5.989	0.008	0.503	0.188–1.346	0.171
Anxiety rating scores	1.038	1.018–1.058	<0.001	1.044	1.019–1.069	0.001
FT3	1.205	1.122–1.295	<0.001	0.969	0.845–1.111	0.651
FT4	1.100	1.066–1.135	<0.001	1.096	1.034–1.162	0.002
TSH	0.778	0.665–0.910	0.002	0.897	0.775–1.039	0.147
TRAb	1.007	0.976–1.038	0.678			
Atherosclerotic disease	1.248	1.195–1.556	<0.001	1.225	1.196–1.365	<0.001
Combine with other drugs	1.677	0.839–3.352	0.143			

GD, Graves' disease; ATD, antithyroid drug; WHO goiter classification system, Grade I, thyroid palpable but not visible when the neck is in the normal position; Grade II, thyroid palpable and visible but within the sternocleidomastoid; Grade III, thyroid palpable and visible but beyond the sternocleidomastoid; FT3, free triiodothyronine; FT4, free thyroxine; TSH, thyrotropin; TRAb, thyrotropin receptor antibody; MMI, Methimazole; OR, odds ratio; CI, confidence interval.

### Prediction of relapse by risk stratification

**Figure 4:**
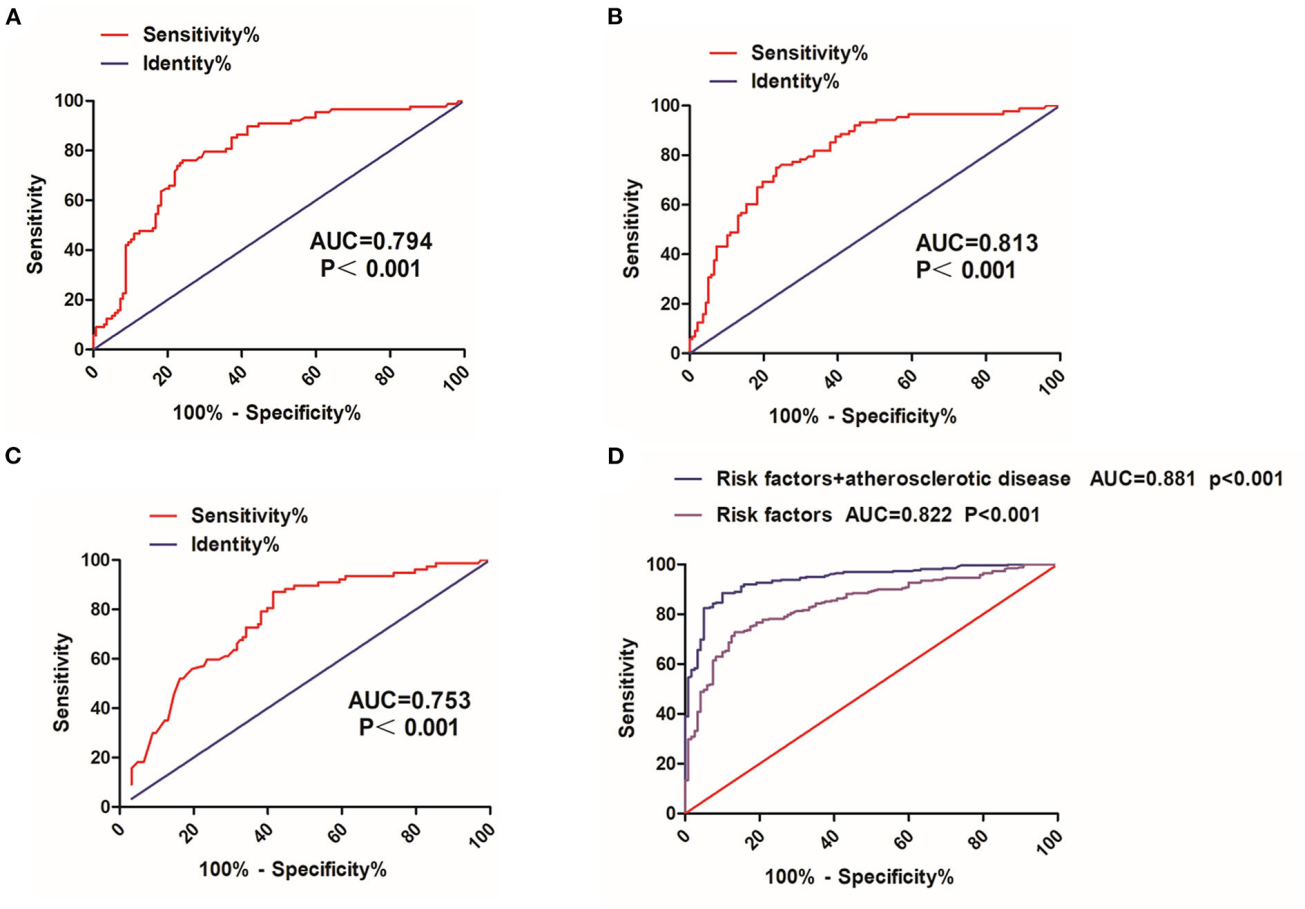
ROC curve analysis of FT3, FT4, and TSH. **(A)** FT3, free triiodothyronine, **(B)** FT4, free thyroxine, and **(C)** TSH, thyrotropin. **(D)** Risk factor (FT3, FT4, and TSH) and atherosclerotic disease ROC curve, receive operating characteristic curve; AUC, the area under the ROC curve.

Given the significant differences in the FT3, FT4, and TSH between two groups, ROC curve analysis was used to evaluate the predictive effects of FT3, FT4, and TSH in distinguishing relapse from remission (Table 5). The area under the ROC curve (AUC) for FT3, FT4, and TSH for remission versus relapse was 0.794 (*P* < 0.001), 0.813 (*P* < 0.001), and 0.753 (*P* < 0.001), respectively (Figures 4A–C). Furthermore, we found that FT3 achieved a maximum sensitivity of 73.86% and specificity of 77.37% for relapse when using a cut off value of 6.585, FT4 achieved a maximum sensitivity of 75.00% and specificity of 76.64% for relapse when using a cut off value of 19.270, TSH achieved a maximum sensitivity of 87.01% and specificity of 58.54% for relapse when using a cut off value of 0.254 (Table 5). Furthermore, we found that combining atherosclerosis significantly increased the predictive ability of FT3, FT4, and TSH for recurrence (Figure 4D).

**Table 5:** Cut of values of FT3, FT4, and TSH.

**Variables**	**Cut off value**	**Sensitivity (%)**	**95%CI**	**Specificity (%)**	**95%CI**	**Likelihood rate**
FT3	>6.585	73.86	63.41–82.66	77.37	69.45–84.08	3.26
FT4	>19.270	75.00	64.63–83.62	76.64	68.66–83.44	3.21
TSH	>0.254	87.01	77.41–93.59	58.54	49.31–67.35	2.10

FT3, free triiodothyronine; FT4, free thyroxine; TSH, thyrotropin; CI, confidence interval.

## Discussion

The major findings of this study can be listed as follows: (1) Elevated goiter gradation Grade II and above, anxiety rating scores and FT4 were found to be independent risk factors of drug reduction relapse, after adjustments for traditional risk factors. (2) Patients with FT4 > 18.82pmol/L (*P* < 0.001), anxiety rating scores > 23 (*P* = 0.007) and Gradation of goiter ≥ Grade II had higher risk of relapse than patients with lower levels. (3) Atherosclerotic disease is closely related to the relapse of hyperthyroidism, ATD treatment in hyperthyroid patients with atherosclerosis should be given more attention.

As a first-line treatment for patients with GD, MMI, a representative drug of ATD, can not only alleviate clinical symptoms of GD but also significantly improve thyroid function. However, there was still evidence of relapse in patients with GD after ATD withdrawal (11–13, 21) and during drug reduction (17–20). Previous studies mainly considered age (<40 years), gender, smoking history, FT3/FT4 ratio at withdrawal, goiter degree, TRAb titer, TSH level (at withdrawal) and duration of treatment were closely associated with relapse after ATD withdrawal (2, 13, 22–24), while there were barely evidence suggested that relapse risk factors during drug reduction. This study retrospectively analyzed the clinical and laboratory characteristics of relapse during drug reduction in patients with GD, and demonstrated that goiter, FT4 and anxiety rating scores were independent risk factors of drug reduction relapse.

Previous studies have demonstrated that goiter and FT4 had a great influence on relapse after ATD withdrawal (13, 25–27). In a multicenter retrospective study of 908 patients with GD followed up for about 10 years, Chung presented that high level of FT4 can be identified as risk factors for relapse after withdrawal of ATD (23), nations and ethnic differences were a plausible explanation for this result. According to this study, high serum level of FT4 were not only independently related with relapse after drug withdrawal, but also strongly associated with relapse during drug reduction. Furthermore, further research revealed that, when FT4 > 18.82 pmol/L, patients with GD had increasingly higher rates of relapse and took less time to achieve relapse. Simultaneously, FT4 > 19.270 pmol/L can accurately predict relapse during drug reduction. In addition, large goiter is a main clinical manifestation of patients with GD (27, 28). Likewise, goiter also strongly correlated with relapse after ATD withdrawal. As far as we know, GD is an autoimmune disorder that TRAb stimulates the thyroid gland and leads to hyperthyroidism. Also, Thyroid lymphoid hyperplasia is regarded as the most common pathological features of patients with GD. Proliferative lymphocytes secrete inflammatory factors to further damage surrounding tissues and increase inflammatory response by TRAb (16, 29). The result of this study confirmed that goiter gradation Grade II and above is closely associated with relapse. Interestingly, it is demonstrated that patients with goiter gradation Grade II and above have a higher recurrence rate and relapse in a relatively short period of time than patients with goiter gradation Grade I. This finding is in agreement with that of Turkish study enrolled 517 patients with GD after ATD withdrawal (30). Meanwhile, there has also been an association between goiter and relapse after ATD withdrawal in previous studies (28, 31). Our risk stratification using this risk factor may be useful when physicians are making a decision on reducing ATD or withdrawal.

Furthermore, drug reduction relapse was also evaluated by pie chart. Intriguingly, reduced dosage of 2.5 mg was assessed the most likely period for relapse. Based on this result, one could speculate that dosage reduction accounted for the largest proportion of current oral ATD drugs during this period. Therefore, the duration of maintenance dose stage should be extended appropriately, and reduced drug should be considered carefully during this period.

Additionally, it is well-known that mental disorders are one of the main causes and complications of GD (32). Patients with GD had a regulatory effect on serotonin and norepinephrine, and the decrease of serotonin and norepinephrine can cause depression and anxiety. Furthermore, the aggravation of GD was also associated with mental disorders (33, 34). Stress and adverse events may impact the immune system, resulting in an inflammatory response characterized by increased white blood cell counts and C-reactive protein (CRP) levels (35, 36). However, the underlying mechanism still remains unclear. As for this study, we confirmed that anxiety rating scores can properly predict recurrence during drug reduction. It is worth noting that, patients with anxiety rating scores > 23 was demonstrated to be a high recurrence rate and relapse early compared to anxiety rating scores < 23 patients. This viewpoint is basically consistent with previous studies (37–39) in relapse after ATD withdrawal. Our study extends the prior findings that physical and mental health is equally vital for patients with GD. Taken together, besides adjusting drug dosage through patients' thyroid function, patients' mental evaluation should also be taken into account during drug reduction.

In addition, there is increasing evidence that atherosclerosis is associated with thyroid dysfunction (40, 41). Gudurn analyzed quantitative coronary CT angiographic parameters and plaque morphology in 774 patients with overt and subclinical hyperthyroidism over a period of 168 months. Indicated that patients with hyperthyroidism have more severe coronary stenosis, plaque burden, and high-risk plaque features, suggesting that elevated thyroid hormones may contribute to coronary vascularization and plaque instability (10). According to this study, we also found that atherosclerotic diseases were significantly higher in the relapsing group which demonstrated that atherosclerotic disease is closely related to the relapse of hyperthyroidism. Here is some indirect evidence to explain this finding. High white blood cell levels and high inflammatory response have been shown to be strongly associated with a poor prognosis for hyperthyroidism (42, 43). As we know, atherosclerosis is a chronic inflammatory disease with persistent release of inflammatory factors from immune cells in plaques, which may increase the progression of hyperthyroidism. Furthermore, patients with atherosclerosis tend to have a combination of metabolic diseases such as hyperemia and diabetes, which may also have a potential impact on hyperthyroidism. Overall, our data reveal for the first time that atherosclerosis-related disease is strongly associated with relapse in patients with hyperthyroidism who discontinue medication. More attention should be paid to treatment of ATD in hyperthyroidism patients with atherosclerosis.

### Study limitations

This study still has several potential limitations. First of all, the retrospective design of the study may have introduced a selection bias. Additionally, this study was a single-center study where all patients were enrolled from the Second Affiliated Hospital of Harbin Medical University. Therefore, considering that the enrolled patients were relatively local, the results may be characterized by regional restrictions. Secondly, we enrolled a relatively small number of patients, including 225 patients with GD which may have affected the results of risk factors during drug reduction. Thirdly, this study only recorded the clinical baseline at the time of the patient's visit, and did not analyze the data during the treatment period, which should be discussed in the follow-up study. Finally, other markers such as blood routine examination, liver function, renal function, ion measurement, and 3D thyroid color Doppler ultrasound or elastic score were not assessed, paving a way for future study to clarify this causal association.

To the best of our knowledge, we demonstrated for the first time an association of serum levels of FT3, FT4, TSH in patients with GD during drug reduction. FT3, FT4, TSH can predict properly for drug reduction to help managing patients with GD clinically.

## Data availability statement

The raw data supporting the conclusions of this article will be made available by the authors, without undue reservation.

## Ethics statement

The studies involving human participants were reviewed and approved by the Second Affiliated Hospital of Harbin Medical University Ethics Committee. The patients/participants provided their written informed consent to participate in this study.

## Author contributions

XZhu and HQ: conception, design, and administrative. YZ and XZhan: support and provision of studymaterials or patients. XZhao and ZR: collection and assembly of data. YW and XY: data analysis and interpretation. BH and XZhao: manuscript writing. All authors approved the final manuscript.

## Funding

This research was supported by the National Natural Science Foundation of China (Nos. 8187120246 and 8167120241).

## Conflict of interest

The authors declare that the research was conducted in the absence of any commercial or financial relationships that could be construed as a potential conflict of interest.

## Publisher's note

All claims expressed in this article are solely those of the authors and do not necessarily represent those of their affiliated organizations, or those of the publisher, the editors and the reviewers. Any product that may be evaluated in this article, or claim that may be made by its manufacturer, is not guaranteed or endorsed by the publisher.
